# A Variable Photo-Model Method for Object Pose and Size Estimation with Stereo Vision in a Complex Home Scene

**DOI:** 10.3390/s23156924

**Published:** 2023-08-03

**Authors:** Hongzhi Tian, Jirong Wang

**Affiliations:** 1 College of Mechanical and Electrical Engineering, Qingdao University, Qingdao 266071, China; wangjirong@qdu.edu.cn; 2 Weihai Innovation Research Institute, Qingdao University, Weihai 264200, China

**Keywords:** pose estimation, photo-model, stereo vision, pixel per metric ratio, genetic algorithm

## Abstract

Model-based stereo vision methods can estimate the 6D poses of rigid objects. They can help robots to achieve a target grip in complex home environments. This study presents a novel approach, called the variable photo-model method, to estimate the pose and size of an unknown object using a single photo of the same category. By employing a pre-trained You Only Look Once (YOLO) v4 weight for object detection and 2D model generation in the photo, the method converts the segmented 2D photo-model into 3D flat photo-models assuming different sizes and poses. Through perspective projection and model matching, the method finds the best match between the model and the actual object in the captured stereo images. The matching fitness function is optimized using a genetic algorithm (GA). Unlike data-driven approaches, this approach does not require multiple photos or pre-training time for single object pose recognition, making it more versatile. Indoor experiments demonstrate the effectiveness of the variable photo-model method in estimating the pose and size of the target objects within the same class. The findings of this study have practical implications for object detection prior to robotic grasping, particularly due to its ease of application and the limited data required.

## 1. Introduction

For home service robots, vision systems are widely used in the perception of environment target objects [1]. Estimating an object’s 6DOF pose and size is important for autonomous robots to track or grasp it. Stereo vision is a widely adopted and low-cost method for estimating a 3D pose. Compared with RGB-D sensing, it perceives a greater variety of target material properties and light conditions [2,3]. However, detecting the 3D pose of arbitrary objects has remained a challenge, particularly when the shape or size of the target object cannot be predetermined.

In terms of the pose detection, stereo-vision methods can be roughly divided into stereo-matching and model-matching methods. Stereo matching, also known as disparity estimation, aims to find the corresponding points of a physical point in a pair of rectified stereo images. Furthermore, through epipolar geometry, stereo vision computes the 3D coordinates of this physical point (2D–3D method). According to the number of matching points, they are divided into feature-based [3] and point-cloud-based methods.

Feature-based methods only match some feature points of the target and take the pose estimation with these points [4,5,6]. Point-cloud-based methods generate a scene point cloud, which can be seen as a global extension of feature-based methods. They use 2D image object detection to segment the corresponding point cloud for pose detection. However, it is generally necessary to organize and structure the 3D discrete points into a higher-level representation, such as voxels [7,8]. Removing mismatched noise points and identifying and segmenting target objects in point clouds are complex problems [9]. However, no matter which method is used, mismatches are inevitable.

Model-based matching methods, also known as template-based methods, can avoid mismatches and are also suitable for occlusion situations [10,11,12]. All the points of a solid 3D model as a group are projected into stereo-vision image planes and are matched with the actual target (3D–2D method). Model generation is a difficult task, relying on the model’s style and size. Some learning-based methods detect objects in a 2D image and then segment the RGB-D point cloud to create a 3D model [13]. However, the size of the models is difficult to change. Several researchers have used deformable models combined with stereo vision to measure the size of tuna with excellent results [14]. However, the complexity of the model building limits the generality of this method in detection.

We have previously proposed a photo-model-based pose estimation method. This method involves segmenting the target object from a photo and constructing a 2D photo-model of it. A 3D photo-model is generated from the 2D photo-model. The pose-changed 3D photo-model is projected onto stereo-vision image planes, and matches are made with the actual target. This process can be summarized as 2D–3D–2D [15]. Experiments have proven the reliability and effectiveness of the photo-model approach for pose estimation using one known distance photo [16].

However, this method required photographing an object of unknown size at a specific distance in order to determine the pixel/metric (PM) ratio. From this ratio, the object’s actual size was calculated and a 2D photo-model generated. We also experimentally demonstrated that the pose of an object can be estimated and tracked in real time [16].

The PM ratio is an important parameter for building a 3D photo-model from the 2D photo with the same size as the object [17,18]. Other studies usually rely on camera calibration with reference objects of known size to ensure this ratio [19,20]. However, suppose the shooting distance of the photos is unknown or there is no reference object; in these cases, they cannot obtain the PM ratio. In the work described in this paper, no special photos are required. The proposed method assumes the PM ratio and converts the 2D photo-model into variable 3D plane photo-models. Through stereo-vision model matching and a genetic algorithm (GA), it can assure the object’s pose and size at the same time.

On the other hand, in our previous studies, 2D photo-model making relied on the threshold segmentation of simple background photos [15,16]. However, the threshold value needed to be reset when the background changed. Due to the development of modern deep learning techniques, object detection in 2D photos has achieved good results in different contexts [21]. This study uses the training results of YOLOv4 [22] on the MS COCO dataset (https://github.com/AlexeyAB/darknet#how-to-evaluate-ap-of-yolov4-on-the-ms-coco-evaluation-server, accessed on 20 September 2022) to detect the object and simplify the 2D photo-model generation process. Size-variable 3D photo-models are generated from a 2D photo by assuming the PM ratio of the pixel length to the actual length of the object. Since the prepared photo does not involve multiple classes, and the production process does not require real-time capabilities, the widely used algorithm YOLOv4 is selected for this purpose [22]. During the experiment, YOLOv8 had not been released yet [23]; thus, it is not utilized in this paper. Additionally, the Transformer algorithm has also demonstrated excellent performance in object detection [24]. However, the main focus of this paper is not on 2D object detection but rather on determining whether the spatial dimensions of the generated photo-models can be used for the pose and size detection of similar objects. In the subsequent experiments, it was found that the YOLOv4 model effectively detected and accurately outlined the objects in the prepared photos.

In terms of 3D pose detection, the proposed variable photo-model method belongs to the model-based matching method not a data-driven method; hence, it requires no additional training [25,26], and it only needs to run on CPUs with limited hardware. Using the similarity factor of the matching degree of the projected model in the left and right images, we constructed a new photo-model matching function. We hope to improve the existing photo-model-based algorithms and lay a good foundation for future research on visual servo systems.

With an industrial product and a piece of fruit, pose–size detection experiments were conducted to verify the effectiveness of the proposed method for daily life. According to the results, with only one category of photo, the target’s pose and size could be estimated.

More precisely, the contributions of this paper are as follows:

(1) This paper allows the utilization of photos taken at unknown distances for model generation. It extends the traditional photo-model-based approach;
(2) With just one photo, this method enables the generation of 3D plane models with varying aspect ratios and sizes, which can be used for object pose estimation;
(3) The variable photo-model method combines deep learning techniques to simplify the traditional algorithm model creation process. It leverages pre-trained weights from existing datasets, eliminating the need for additional training. One of its advantages is that it can be executed on a CPU with limited hardware resources.

The rest of the present paper is organized into the following sections: Section 2 provides an overview of the relevant literature and previous studies. Section 3 presents variable photo-model generation and the photo-model pose and size estimation method. In Section 4, we discuss the adaptability of the proposed method for recognizing an object’s pose and size according to the experimental results. The conclusions and future work are described in Section 5.

## 2. Related Work

Regarding partial occlusion, several previous studies [15,27] have explored different environmental factors affecting its handling. These studies provide experimental evidence to support the effectiveness of the photo-model approach [15,27].

Furthermore, in handling different lighting conditions, the practicality of the photo-model-based method was tested experimentally [28]. The experiments focused on two common light sources: fluorescent and light-emitting diode (LED) lighting. The method’s ability to tolerate changes in illumination for object recognition was analyzed, and the results demonstrated its robustness in handling different light sources and levels of illumination. Additionally, a visual servo system was developed for capturing marine creatures [29]. The adaptability of the photo-model method to these factors will not be discussed further in this article.

On the other hand, research on 3D indoor object detection using stereo images is still limited. There is a model-based approach that utilizes object model projections on synthetic and real datasets to train networks to detect object poses [30]. However, most existing datasets for pose estimation rely on RGB-D data rather than binocular vision [31]. Furthermore, while there have been studies exploring the use of infrared (IR) stereo imaging for vegetable classification [32], the available stereo benchmark datasets primarily consist of RGB imagery and lack object size information. This lack of comprehensive benchmark datasets has led many studies in stereo vision pose estimation to rely on their own target-specific datasets instead of publicly available benchmarks [33,34]. As a result, it is common for researchers in the field of stereo vision pose estimation to utilize their own datasets.

In the next section, related work in the field of photo-model-based methods and object pose detection is reviewed. The limitations of existing databases are also discussed. Regarding 3D pose detection, the variable photo-model method belongs to the model-based matching approach and does not rely on extensive data-driven techniques [25,26,34]. This eliminates the need for additional training and allows the method to run efficiently on CPUs with limited hardware resources. The approach combines both deep learning techniques and traditional methods.

## 3. Variable Photo-Model Pose and Size Detection Method

This section introduces the variable photo-model pose and size detection methodology.

**Figure 1:**
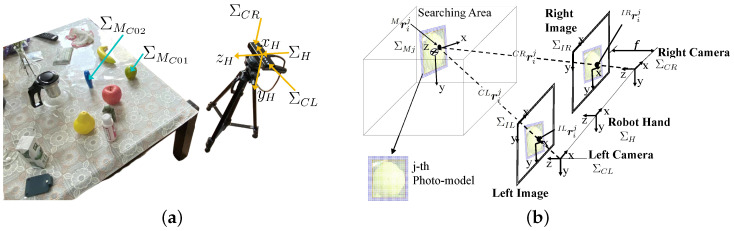
(**a**) Experimental environment and related coordinate systems. (**b**) Perspective projection of a photo-model of a pear in the stereo vision system. In the 3D search space, the spatial plane model is projected onto the left and right images through perspective projection.

Figure 1a shows the experimental environment. Each coordinate system is as follows:

- $\Sigma_{H}$: end-effector (hand) coordinate system;
- $\Sigma_{M_{C01}},\Sigma_{M_{C02}}$: target object coordinate system;
- $\Sigma_{CL}$, $\Sigma_{CR}$: left and right camera coordinate systems.

Figure 1b shows a perspective projection of the stereo vision system. Each coordinate system is as follows:

- $\Sigma_{IL},\Sigma_{IR}$: left and right image 2D coordinate systems;
- $\Sigma_{Mj}$: *j*-th model coordinate system;
- ${}^{M}r_{i}^{j}$: position of the *i*-th point on the *j*-th 3D model in $\Sigma_{Mj}$;
- ${}^{CL}r_{i}^{j}$, ${}^{CR}r_{i}^{j}$: position of the *i*-th point on the *j*-th 3D model based on $\Sigma_{CR}$ and $\Sigma_{CL}$;
- ${}^{IL}r_{i}^{j}$, ${}^{IR}r_{i}^{j}$: projected 2D position on $\Sigma_{IL}$ and $\Sigma_{IR}$ of the *i*-th point on the *j*-th 3D model.

### 3.1. Variable Photo-Model Generation

This subsection describes the model generation before explaining the stereo-matching method. The model generation has two central parts. The first part is to generate a fixed 2D pixel model in pixel units. The latter is a 3D plane model generation; its size (length and width) in millimeters is variable. Estimation of the relative pose requires the use of the generated 3D planar model.

**Figure 2:**
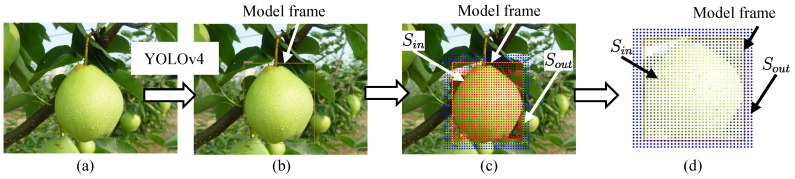
Model generation processes are described as: (**a**) one downloaded photo with the same type as the target. Its size is 1066 × 799 pixels. (**b**) A bounding box detected by YOLOv4 is defined as the model frame. (**c**) The model is composed of the inner portion $S_{in}$ and outer portion $S_{out}$ with sampling points. (**d**) The generated model. The model is only a small part of the photo including the target, the whole photo is not a model. Sampling points are collected at a certain interval.

**Figure 3:**
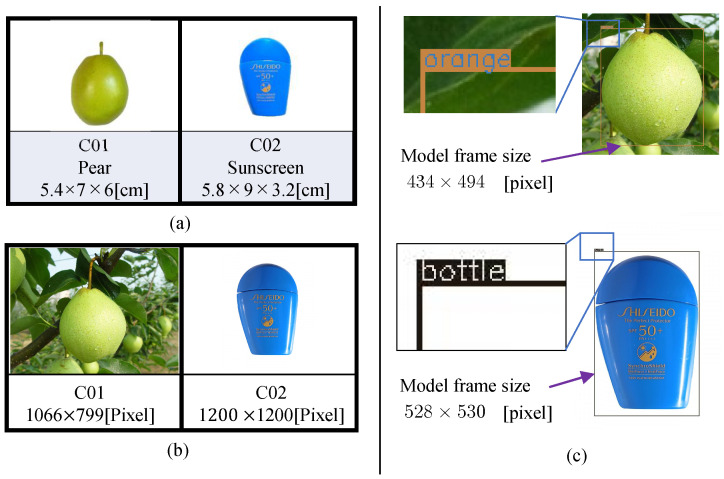
(**a**) Two objects. Code names are C01 and C02. The three labels correspond to model number, English name, and size. (**b**) Prepared photos downloaded from Bing Images (https://cn.bing.com/images, accessed on 29 September 2022). The pixel size of each photo is shown at the very bottom of each frame. (**c**) Photo-model frames detected by YOLOv4. The detection boxes represent the interior portion of the photo-model, which is only part of the photo. False detection of the target name does not affect pose detection.

**Figure 4:**
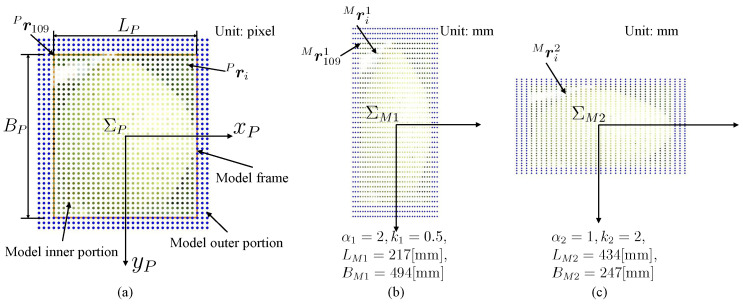
Model display. (**a**) 2D photo-model generated by the process in Figure 2. Its coordinate system $\Sigma_{P}$ is in pixels. The model frame size, i.e., 2D pixel photo-model size, is $L_{P}\times B_{P}=434\times 494$ pixels. (**b**,**c**) The variable 3D plane photo-models. $\beta_{1}=\alpha_{1}k_{1}=1$, $\beta_{2}=\alpha_{2}k_{2}=2$.

Figure 2 shows the model generation process. We did not take a photo of the target pear, but downloaded one photo (Figure 2a) from Bing Images. Figure 3a shows the actual target. Furthermore, Figure 3b shows the downloaded photos. The pre-trained YOLOv4 weight in the existing MS COCO dataset is used to detect the object in the photo. The bounding box is defined as the model frame (Figure 2b). Figure 4a shows the coordinate system of the model $\Sigma_{P}$. The size of the 2D model frame is $L_{P}\times B_{P}$ pixels, i.e., the 2D photo-model pixel size. The outer portion’s size is larger than the model frame size. Sampling points are taken in the model at a regular pixel interval (Figure 2c). The coordinate of the *i*-th sampling point in the 2D pixel coordinate system in $\Sigma_{P}$ is

(1) $${}^{P}r_{i}={{[}^{P}x_{i},{}^{P}y{}_{i}]}^{T}.$$

In order to explore the object, the photo-model needs to be converted from a 2D pixel model to a 3D spatial plane model. The coordinate of the *i*-th point of the *j*-th model ${}^{M}r_{i}^{j}$ in coordinate system $\Sigma_{Mj}$ in 3D searching space is

(2) $${}^{M}r_{i}^{j}={{[}^{M}x_{i}^{j},{{}^{M}y}_{i}^{j},{{}^{M}z}_{i}^{j}]}^{T}={\left[{}^{M}x_{i}^{j},{}^{M}y_{i}^{j},0\right]}^{T}.$$

As shown in Figure 1b, Equation (3) indicates the conversion relationship of the *i*-th sampling point between $\Sigma_{P}$ in Figure 4 and $\Sigma_{Mj}$ in Figure 1b.

(3) $$\begin{array}{c} \left\{\begin{array}{cc} {}^{M}x_{i}^{j}\left(\alpha_{j}\right)\;\left[\mathrm{mm}\right] & =\frac{{}^{P}x_{i}\;\left[\mathrm{pixel}\right]}{\alpha_{j}}\\ {}^{M}y_{i}^{j}\left(\beta_{j}\right)\;\left[\mathrm{mm}\right] & =\frac{{}^{P}y_{i}\;\left[\mathrm{pixel}\right]}{\beta_{j}}, \end{array}\right. \end{array}$$

where

- $\alpha_{j}$: PM ratio of the *j*-th model in the x direction;
- $\beta_{j}$: PM ratio of the *j*-th model in the y direction [20].

The PM ratio unit is (pixel/mm). It is the ratio of the 2D pixel model to the 3D spatial plane model. $\alpha_{M},\beta_{M}$ are defined as the real ratio of the 2D pixel model to the target object. The relationship between $\alpha_{j}$ and $\beta_{j}$ is

(4) $$\beta_{j}=\alpha_{j}k_{j},$$

where $k_{j}$ is the ratio factor.

For instance, in Figure 4, at the moment when i=109 and j=1, the calculations are as follows: ${}^{M}x_{109}^{1}\left(\alpha_{1}\right)=\frac{{}^{P}x_{109}}{\alpha_{1}}=\frac{-434/2\;\left[\mathrm{pixel}\right]}{2}=-434\;\left[\mathrm{mm}\right]$, and ${}^{M}y_{109}^{1}\left(\beta_{1}\right)=\frac{{}^{P}y_{109}}{2\times 0.5}=\frac{-494/2\;\left[\mathrm{pixel}\right]}{1}=-247\;[\mathrm{mm}].$

For the *j*-th 3D spatial plane model, its length and width are calculated as in Equation (5).

(5) $$\begin{array}{c} \left\{\begin{array}{cc} L_{Mj}\;\left[\mathrm{mm}\right] & =\frac{L_{P}\;\left[\mathrm{pixel}\right]}{\alpha_{j}}\\ B_{Mj}\;\left[\mathrm{mm}\right] & =\frac{B_{P}\;\left[\mathrm{pixel}\right]}{\beta_{j}}. \end{array}\right. \end{array}$$

Equation (5) converts the 2D pixel model into a 3D spatial plane model. The thickness of the model is ${}^{M}z_{i}=0$; therefore, the resulting 3D photo-model is a 3D space plane. In this study, ${}^{M}r_{i}^{j}$ is developed and can be described as the function of $\alpha_{j},k$, i.e., ${}^{M}r_{i}^{j}\left(\alpha_{j},k_{j}\right)$.

The 3D plane model is composed of dots whose relative positions are predefined as in Figure 4.

### 3.2. Projective Transformation of the Photo-Model

The projective transformation of the fixed photo-model has been proposed in our previous paper [16,35]. In the past, since ${}^{M}r_{i}^{j}$ is generated from the original object’s photo, it is a size-fixed model, and its size is the same as the real target. In this paper, ${}^{M}r_{i}^{j}$ is a variable photo-model, and thus a function of the PM ratio.

As shown in Figure 1a, the pose of $\Sigma_{M_{C01}}$ based on $\Sigma_{H}$, including three position variables and three orientation variables in quaternion [16], is

(6) $${}^{H}\phi_{M}=\left[{{}^{H}x}_{M},{{}^{H}y}_{M},{{}^{H}z}_{M},{{{}^{H}\varepsilon }_{1}}_{M},{{}^{H}\varepsilon_{2}}_{M},{{}^{H}\varepsilon_{3}}_{M}\right]{}^{T}.$$

As shown in Figure 1b, based on $\Sigma_{H}$, the pose of the *j*-th 3D model ${}^{H}\phi_{M}^{j}$ is defined as

(7) $${}^{H}\phi_{M}^{j}={{[}^{H}x_{M}^{j},{}^{H}y_{M}^{j},{}^{H}z_{M}^{j},{}^{H}\varepsilon_{1M}^{j},{}^{H}\varepsilon_{2M}^{j},{}^{H}\varepsilon_{3M}^{j}]}^{T},$$

which has been explained in previous studies [16,35].

For simplicity, ${{}^{H}\phi }_{M}^{j}$ is written as ${{}^{}\phi }_{M}^{j}$. The homogeneous transformation ${}^{H}T_{Mj}^{}$, based on the hand coordinate system $\Sigma_{H}$, can be calculated through the pose of the *j*-th model ${{}^{}\phi }_{M}^{j}$ [36].

Concerning stereo vision, position ${}^{CL}r_{i}^{j}$ of the *i*-th point based on $\Sigma_{CL}$ can be calculated through Equation (8),

(8) $${}^{CL}r_{i}^{j}=\;\;{}^{CL}T_{H}{}^{H}T_{Mj}\left(\phi_{M}^{j}\right)\;\;{}^{M}r_{i}^{j}\left(\alpha_{j},k_{j}\right).$$

On the *j*-th 3D model using the projective transformation matrix $P_{CL}$, ${}^{CL}r_{i}^{j}$ is projected from 3D space $\Sigma_{CL}$ into 2D left image space $\Sigma_{IL}$ as

(9) $$\begin{array}{ccc} {}^{IL}r_{i}^{j} & = & P_{CL}{}^{CL}r_{i}^{j}\\ & = & P_{CL}{}^{CL}T_{H}{}^{H}T_{Mj}\left(\phi_{M}^{j}\right){}^{M}r_{i}^{j}\left(\alpha_{j},k_{j}\right). \end{array}$$

Then ${}^{IL}r_{i}^{j}$ can be described in short as

(10) $$\begin{array}{c} {}^{IL}r_{i}^{j}=f_{L}\left(\Phi_{M}^{j}\right), \end{array}$$

where

(11) $$\Phi_{M}^{j}={\left[\phi_{M}^{Tj},\alpha_{j},k_{j}\right]}^{T}.$$

**Figure 5:**
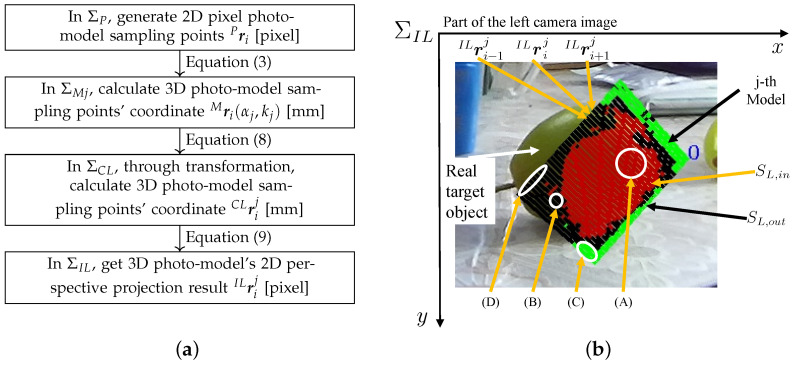
(**a**) The summary of the calculation process from the photo-model generation to the model’s stereo vision perspective projection (2D–3D–2D). The key node formulas are pointed out. (**b**) A model matching result. ${}^{IL}r_{i}^{j}$ is the *i*-th sampling point’s coordinate of the *j*-th model in the left image $\Sigma_{IL}$. Sampling points are given a positive or negative score. (A) The sampling points inside the model overlap with the real target to acquire a positive score $p_{L,in}^{ij}>0$. (B) Model internal sampling points overlap with the background to acquire negative values $p_{L,in}^{ij}<0$. (C) The outer portion overlaps with the background $p_{L,out}^{ij}>0$. Furthermore, (D) shows that the outer portion overlaps with the real target $p_{L,out}^{ij}<0$.

${}^{IR}r_{i}^{j}$ can also be described in the same manner as ${}^{IL}r_{i}^{j}$. The projective transformation process is summarized in Figure 5a, i.e., 2D–3D–2D [15]. The projection calculation process of the C02 photo-model is the same as that of C01. The series of equations from Equations (1) to (10) presents a detailed and systematic procedure for a 2D–3D–2D process. This process begins by generating a 3D photo-model utilizing a single photo, culminating in mapping pose transformations to dual eye images.

### 3.3. Photo-Model Matching and Spatial Fitness Function

In Figure 1b, through the forward projection Equation (10), a generated 3D planar model ${}^{M}r_{i}^{j}$ is projected from the 3D search space onto the left and right camera images. Figure 5b is the actual left image projection example. The projection results of the inner $S_{in}$ and outer $S_{out}$ parts of the model in the left image are $S_{L,in}$ and $S_{L,out}$. The projection process for the right image is similar to the left image. Furthermore, the model projection results are of $S_{R,in}$ and $S_{R,out}$.

The HSV color representation is used for the extraction of the target color (Figure 2d). The advantage of HSV is that each of its attributes correspond directly to the basic color concepts, which makes it conceptually simple. In addition, the hue of the HSV color system shows good robustness against a change in the lighting intensity.

The fitness function is defined as an evaluation of how well the projection model matches the real target in images captured by the binocular camera, i.e., the similarity measurement.

**Figure 6:**
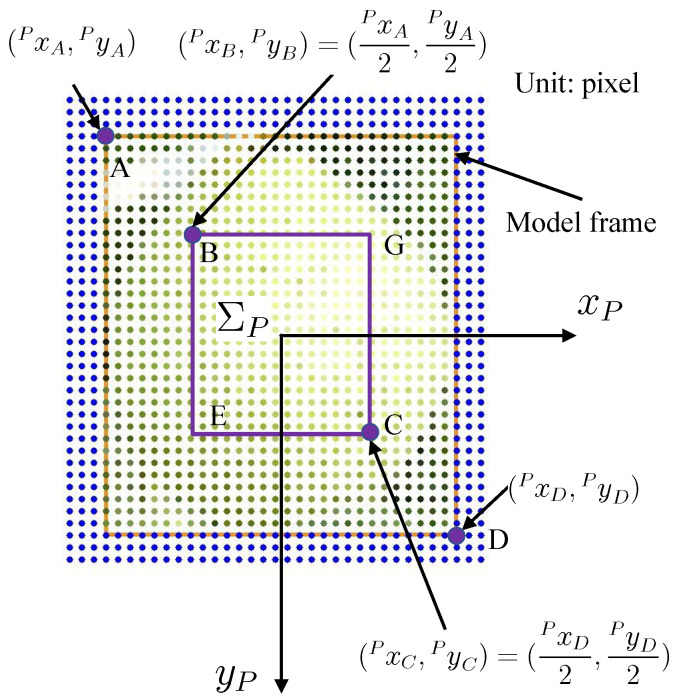
The average hue of the sampling points in the inner rectangle BECG is used as the evaluation threshold for the addition or subtraction of $p_{L,out}^{ij}$ and $p_{R,out}^{ij}$ of the outer point.

The symbols related to function computation are explained as follows:

$C_{IL}^{ij},C_{IR}^{ij}$:  the color of point ${}^{IL}r_{i}^{j}$ or ${}^{IR}r_{i}^{j}$ on the captured left and right images, and the judgment conditions with HSV ($H_{IL}^{ij}$, $S_{IL}^{ij}$, $V_{IL}^{ij}$, $H_{IR}^{ij}$, $S_{IR}^{ij}$, $V_{IR}^{ij}$) are shown in Table 1;
$C_{ML}^{ij}$:  the stored color of the *i*-th point of the *j*-th model (Figure 4), and the judgment conditions ($H_{ML}^{ij}$, $S_{ML}^{ij}$, $V_{ML}^{ij}$) are shown in Table 1;
$p_{L,in}^{ij},p_{L,out}^{ij}$:  evaluation of the sampling point inside and outside the model frame in the left image;
$p_{R,in}^{ij},p_{R,out}^{ij}$:  evaluation of the sampling point inside and outside the model frame in the right image;
$N_{in},N_{out}$:  the total number of the inner and outer portion sampling points;
${\bar{H}}_{in}$:  The average hue of the sampling points in the rectangle BECG in Figure 6. This is used as the evaluation threshold for the addition or subtraction $p_{L,out}^{ij}$ and $p_{R,out}^{ij}$ of the outer point;
$e_{1},e_{2}$:  Evaluation value of a sampling point in the inner portion. $e_{1}=2$, $e_{2}=-0.5$, These evaluation values are tuned experimentally;
$e_{3},e_{4}$:  Evaluation value of a sampling point in the outer portion. $e_{3}=0.5$, $e_{4}=-1.9$. These evaluation values are tuned experimentally.

Equations (12) and (13) are the designed fitness between the target captured by stereo cameras and the projected *j*-th model on the left and right images, respectively, [16].

(12) $$\begin{array}{c} F_{L}^{j}\left(\Phi_{M}^{j}\right)=(\;\sum_{\begin{array}{c} {}^{IL}r_{i}^{j}\in \\ S_{L,in} \end{array}}\;p_{L,in}^{ij}+\;\sum_{\begin{array}{c} {}^{IL}r_{i}^{j}\in \\ S_{L,out} \end{array}}\;p_{L,out}^{ij})/m. \end{array}$$

(13) $$\begin{array}{c} F_{R}^{j}\left(\Phi_{M}^{j}\right)=(\sum_{\begin{array}{c} {}^{IR}r_{i}^{j}\in \\ S_{R,in} \end{array}}p_{R,in}^{ij}+\sum_{\begin{array}{c} {}^{IR}r_{i}^{j}\in \\ S_{R,out} \end{array}}p_{R,out}^{ij})/m. \end{array}$$

In a single image, left or right, the theoretical maximum fitness of the projected *j*-th model is

(14) $$m=e_{1}N_{in}+e_{3}N_{out.}$$

Equations (15) and (16) are used to calculate $p_{L,\mathrm{in}}^{ij}$ and $p_{L,\mathrm{out}}^{ij}$, respectively, which are included in Equation (12) as proposed previously [16].

(15) $$p_{L,in}^{ij}=\left\{\begin{array}{cc} e_{1}, & \mathrm{if}C_{IL}^{ij}\;\mathrm{and}\;C_{ML}^{ij}\;\mathrm{are}\;\mathrm{close};\\ e_{2}, & \;\mathrm{otherwise},\; \end{array}\right.$$

(16) $$p_{L,out}^{ij}=\left\{\begin{array}{cc} e_{3}, & \mathrm{if}(|H_{IL}^{ij}-{\bar{H}}_{in}|⩾15);\\ e_{4}, & \mathrm{if}(|H_{IL}^{ij}-{\bar{H}}_{in}|<15). \end{array}\right.$$

**Figure 7:**
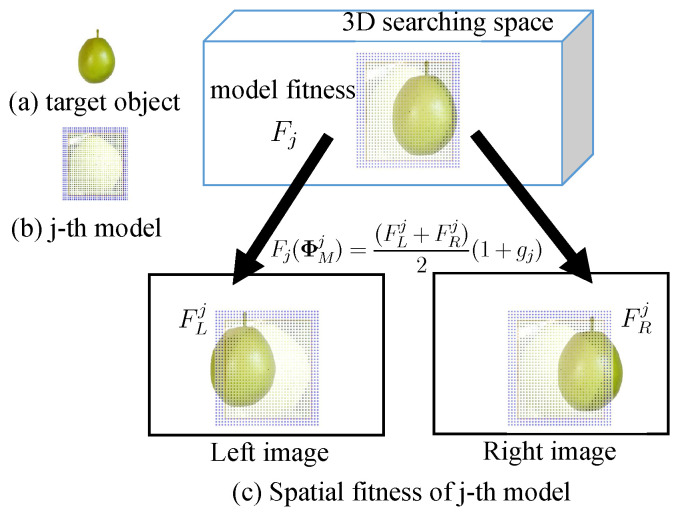
Fitness calculation process. A photo-model in the 3D searching space on the top of this figure is a 3D photo-model with pose and size $\Phi_{M}^{j}$. The left and right 2D searching models represented on the left/right bottom, are calculated by forward projection in Equation (10).

**Figure 8:**
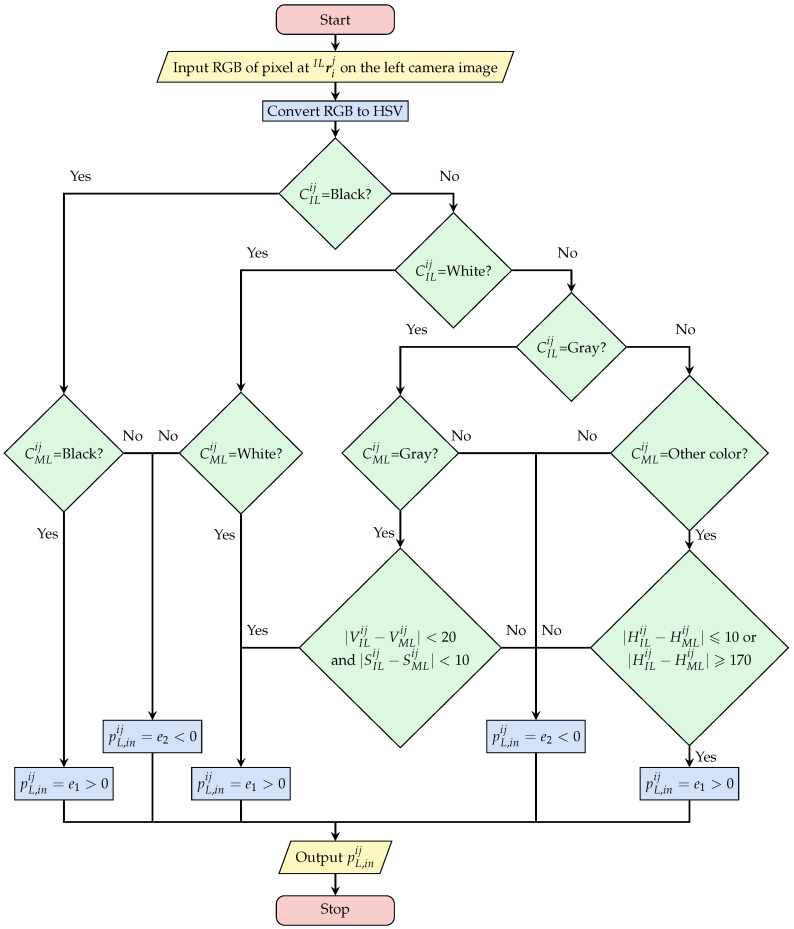
Calculation process of the evaluation value $p_{L,in}^{ij}$ for an internal sampling point. The algorithm complexity of this part is O(1).

Figure 7 shows a generated photo-model placed in the 3D searching space and the left and right 2D searching models that are projected, respectively, from photo-model with the pose and size being assumed to be $\Phi_{M}^{j}$. Figure 8 illustrates the calculation process of the evaluation value $p_{L,in}^{ij}$ for the inner sampling point, including the color judgment process for $C_{IL}^{ij}$ and $C_{ML}^{ij}$ of one inner point. This is a continuous judging process [37].

We divide the colors into four categories: black, white, gray, and other for similarity judgment. For grayscale, it is necessary to judge whether the sampling point color $C_{ML}^{ij}$ is close to the point color $C_{IL}^{ij}$ in the captured image with S and V. For other colors, we only compare their H values.

The algorithm complexity for determining the evaluation value of each individual sampling point (*i*-th point) based on color similarity is considered constant, with a time complexity of O(1). Therefore, the algorithm complexity of Figure 8 can be regarded as O(1). For each photo-model (*j*-th photo-model), the fitness calculation complexity in Equation (12) is O($N_{in}+N_{out}$).

**Table 1:** Color component range according to the OpenCV HSV range. H, S, and V are all integers.

	Black	Gray	White	Other Colors
$H_{min}$	0	0	0	0
$H_{max}$	180	180	180	180
$S_{min}$	0	0	0	31
$S_{max}$	255	30	30	255
$V_{min}$	0	46	221	46
$V_{max}$	45	220	255	255

Figure 6 shows the average hue ${\bar{H}}_{in}$ of the sampling points in the inner rectangle BECG, which is used as the evaluation threshold of the outer portion sampling point $p_{L,out}^{ij}$ or $p_{R,out}^{ij}$.

Figure 5b shows the *j*-th model by 3D to 2D projection on the left image. The coordinates of the sampling points are indicated as ⋯,${}^{IL}r_{i-1}^{j}$, ${}^{IL}r_{i}^{j}$, ${}^{IL}r_{i+1}^{j}\cdots$. In Equation (15) and Figure 8, if the color $C_{IL}^{ij}$ of each point of the captured images, which lies inside the surface model frame $S_{L,in}$, is similar to the color $C_{ML}^{ij}$ of each point in a model, the fitness value will increase with the voting value of $e_{1}$. These sampling points are represented by dots designated by (A) in Figure 5b. The fitness value will decrease with the value of $e_{2}$ for every model inner portion point when $C_{ML}^{ij}$ is different from $C_{IL}^{ij}$ in the left camera image. This represents that the model does not precisely overlap the target in the input image, represented by (B) in Figure 5b.

Similarly, in Equation (16), if $H_{IL}^{ij}$ of a point in $S_{L,out}$ in the left camera image is different with the average hue ${\bar{H}}_{in}$ of the target, with a tolerance of 20, the fitness value will increase with the value of $e_{3}$. This means the $S{\;}_{L,out}$ strip area surrounding $S{\;}_{L,in}$ overlaps with the background, expressing the model and the target overlap correctly as (C) in Figure 5b. Otherwise, the fitness value will be decreased with the value of $e_{4}$. This represents points on $S{\;}_{L,out}$ that overlaps with the real target as (D) in Figure 5b.

Likewise, functions $p_{R,in}^{ij}$ and $p_{R,out}^{ij}$ are calculated in the right camera image.

As shown in Figure 7, to minimize the adverse effect of the high model matching values on pose detection in a single-sided image, a similarity factor is proposed in this study. This factor, denoted as $g_{j}$, is designed as follows:

(17) $$g_{j}=\left\{\begin{array}{cc} 0, & \mathrm{if}\;(F_{L}^{j}=0\;\mathrm{or}\;F_{R}^{j}=0);\\ e^{-{\left(F_{L}^{j}/F_{R}^{j}-\mu \right)}^{2}/2\sigma^{2}}, & \mathrm{otherwise}, \end{array}\right.$$

where μ=1 and σ=0.08. The value of $g_{j}$ is limited to the range [0,1]. Higher values of $g_{j}$ indicate closer values of $F_{L}^{j}$ and $F_{R}^{j}$.

In the end, the stereo matching fitness of the *j*-th model is calculated as

(18) $$F_{j}\left(\Phi_{M}^{j}\right)=\frac{\left(F_{L}^{j}+F_{R}^{j}\right)}{2}\left(1+g_{j}\right).$$

## 4. Pose-Size Estimation Experiment with the Genetic Algorithm

Figure 1a shows the experimental environment. The stereo camera is a ZED 2i. The resolution of the stereo images is 1920×1080 pixels. The PC is a Lenovo Legion Y70002021 (CPU: i5-11400H, 2.70 GHz; RAM: 16 GB).

**Figure 9:**
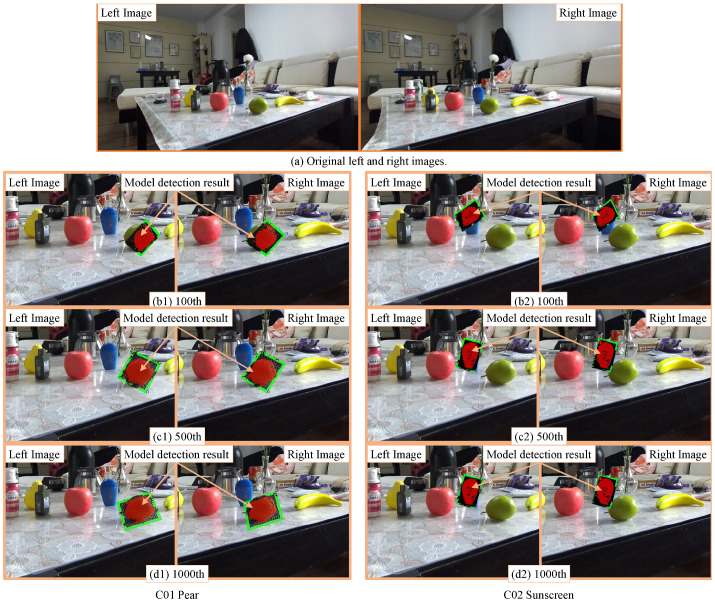
The 3D pose estimation results of the GA. (**a**) shows the original stereoscopic image. (**b1**,**c1**,**d1**) show the magnifying view of the 100th, 500th, and 1000th generation GA exploration results of the target pear. (**b2**,**c2**,**d2**) are the results for the sunscreen.

A pose and size detection experiment was conducted in a real application scenario. Figure 9a shows the images observed by the stereo camera. Using the same left and right photos (Figure 9a), two separate experiments were conducted, each with only one target, a pear and a sunscreen.

The fitness function $F_{j}\left(\Phi_{M}^{j}\right)$ transforms the detection problem into an optimization problem of the pose and ratio $\Phi_{M}^{j}$ [16]. We choose the GA as an optimization method to find the maximum fitness value because of its simplicity and effectiveness [16,38]. According to the GA, the 3D models with random poses and ratios generated from the prepared photos converge to target objects in 3D space. Te GA stops evolving after the 1000th generation.

As shown in Equation (19), each chromosome comprises eight variables. The first three variables $\left({{}^{H}x}_{M}^{j},{{}^{H}y}_{M}^{j},{{}^{H}z}_{M}^{j}\right)$ are the *j*-th model’s position in 3D space, and the middle three variables $\left({{{}^{H}\varepsilon }_{1}^{j}}_{M},{{}^{H}\varepsilon_{2}^{j}}_{M},{{}^{H}\varepsilon_{3}}_{M}^{j}\right)$ are the orientation based on $\Sigma_{H}$. The last variables are the PM ratio $\alpha_{j}$ and factor $k_{j}$.

(19) $$\overset{{{}^{H}x}_{M}^{j}}{\underset{10bits}{\overset{︷}{\underset{︸}{01\cdots 1}}}}\overset{{{}^{H}y}_{M}^{j}}{\underset{10bits}{\overset{︷}{\underset{︸}{00\cdots 1}}}}\overset{{{}^{H}z}_{M}^{j}}{\underset{10bits}{\overset{︷}{\underset{︸}{11\cdots 0}}}}\overset{{{}^{H}\varepsilon }_{1M}^{j}}{\underset{7bits}{\overset{︷}{\underset{︸}{01\cdots 0}}}}\overset{{{}^{H}\varepsilon }_{2M}^{j}}{\underset{7bits}{\overset{︷}{\underset{︸}{01\cdots 1}}}}\overset{{{}^{H}\varepsilon }_{3M}^{j}}{\underset{7bits}{\overset{︷}{\underset{︸}{01\cdots 0}}}}\overset{\alpha_{j}}{\underset{5bits}{\overset{︷}{\underset{︸}{11\cdots 0}}}}\overset{k_{j}}{\underset{5bits}{\overset{︷}{\underset{︸}{01\cdots 1}}}}$$

**Figure 10:**
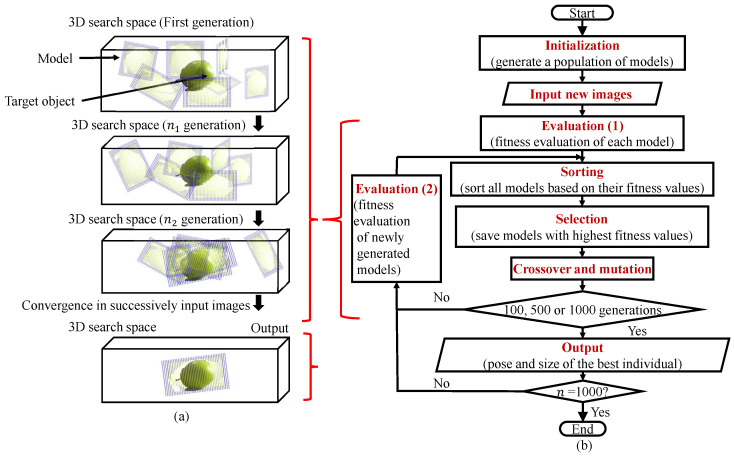
GA evolution process in which 3D models with random poses converge to the real 3D solid target object in 3D space. The pose of the model with the highest fitness value represents the estimated pose of the target object at that instant: (**a**) schematic diagram of the evolutionary process, and (**b**) operational process of GA from “Initialization” to “Output” step by step.

Figure 10 shows a flowchart for the GA evolution process for recognition and pose estimation:

(1) Firstly, the individuals are randomly generated in the 3D searching area as the first generation;
(2) New images captured by dual-eye cameras are input;
(3) The fitness value of every individual is calculated;
(4) Every individual’s fitness value is sorted by the calculated fitness value;
(5) The best individual is selected from the current population, and the weak individuals are removed;
(6) Then, the individuals for the next generation are reproduced by performing crossover and mutation between the selected individuals;
(7) Only new individuals in the next generation are evaluated by the fitness function, shown in “Evaluation (2)” block, because the right and left images do not change and the top individuals with the highest fitness do not need to calculate fitness again since the image is constant;
(8) The above process is repeated until the desired generation is reached. Finally, the GA outputs the best individuals of the 100th, 500th, and 1000th generation, and then terminates the evolutionary process.

Table 2 and Table 3 present a summary of the GA estimation results at different generations, providing the pose $\phi_{M}^{j}$ and size $L_{Mj}\times B_{Mj}$ data of the best fit individuals represented by $F_{j}\left(\Phi_{M}^{j}\right)$. The “Measure” row corresponds to the actual sizes and positions of the targets, which were measured using a manual tape measure. By the 1000th generation, the experimental results closely matched the actual values. The detected object’s pose, length, and width exhibited a close resemblance to their actual counterparts. It is worth noting that the unitless orientation in quaternion represents the pose, and the actual orientation of the targets remains unknown.

In Table 2, the last row shows the distance and size relative errors. From the table, we can observe that the distance error $e_{zC01}$ is less than 2 cm. In Table 3, we can observe that the distance error $e_{zC02}$ is also less than 2 cm.

The measurement results for the sunscreen (Table 3) outperform those for the pear (Table 2). Although the datasets are different [32,39], there are still comparable aspects in terms of object size and pose detection. The pear’s results demonstrate slightly lower accuracy compared to the measurements reported in [32]. On the other hand, the sunscreen’s results exhibit better performance than the corresponding distance measurements presented in [32], despite the lack of object pose detection in that study. Notably, it is worth noting that the pose errors for both objects are similar to the results highlighted in [39].

For both the sunscreen and the pear, the distance *z* detection error is less than 2 cm. Table 3 shows that the GA has already found an optimal solution in the 500th generation, which is the same as in the 1000th generation. This indicates that the algorithm has successfully converged to the best possible solution. Regarding the pear in Table 2, the orientation $\varepsilon_{3}$ at the 1000th generation is −0.77, which is less than −0.5 and indicates a reverse rotation around the $Z_{M}$ axis of more than 90 degrees. However, the actual pose of the pear is lying horizontally and only rotated by less than 90 degrees. The pose detection result is close to the actual pose.

The comparison with other methods is shown in Table 4. Orientation errors are transformed from quaternion to Euler angles ($e_{1}$, $e_{2}$, $e_{3}$) for comparison. Qualitative analysis was performed as above on the pear orientation detection. In general model-based methods, it is assumed that the model has the same size as the object, resulting in no size errors ΔL and ΔB [30,39]. For comparison, we examined findings related to the PM ratio [20] or stereo vision [32] for size measurements, although these studies did not perform pose measurements. Our method can be regarded as comparable to other reliable methods in terms of size and pose measurements. On average, it falls into the upper middle level of accuracy. Furthermore, our method is capable of reliably estimating both size and pose.

Through the experimental results, it is confirmed that:

(1) The proposed variable photo-model-based recognition method utilizes stereo vision and a 2D photo to estimate the pose of a 3D target object, extending the traditional approach;
(2) This method can generate 3D plane models with varying aspect ratios and sizes using just one photo, enabling accurate object pose estimation;
(3) The variable photo-model method combines deep learning techniques, utilizing pre-trained weights from existing datasets, and can be executed on a CPU with limited hardware resources.

## 5. Conclusions and Future Work

The study presented a pose and size estimation method using the variable photo-model. The experimental results using two different objects demonstrated that the generated variable PM ratio photo-model was able to detect the objects’ pose and size in a complex home environment. The accuracy was found to be better for the sunscreen compared to the pear. The adaptability of the variable photo-model method to different target shapes was also observed when using a photo from the same category.

The fact that the detection performance is better for industrially manufactured products (sunscreen) with fixed shapes compared to an agricultural product (pear) with irregular shape variations suggests that the method’s ability to handle shape variations is not sufficiently refined and requires improvement.

In terms of future research, it is recommended to include a wider variety of experimental objects to enhance the generalizability of the findings. Moreover, conducting information extraction from existing datasets for comparative studies would provide valuable insights. Furthermore, the impact of different deep learning models on the generation of photo-models should be thoroughly investigated and analyzed.

## Figures and Tables

**Table 2:** Pear C01 GA’s detection results. Through perspective transformation, the projection results of the model on the left and right images are shown in Figure 9(b1–d1). The table’s “Measure” row shows the target’s measurement under the tape measure.

Generation	*x* [mm]	*y* [mm]	*z* [mm]	$\varepsilon_{1}$	$\varepsilon_{2}$	$\varepsilon_{3}$	α	β=αk	$L_{\mathrm{Mj}}$ [mm]	$B_{\mathrm{Mj}}$ [mm]	$F_{j}(\Phi_{M}^{j})$
100	69	113	542	0.28	−0.15	−0.38	6.94	6.81	63	73	0.5409
500	61	109	556	−0.29	0.14	0.25	6.63	6.38	66	77	0.7527
1000	59	116	558	−0.28	−0.12	−0.77	6.63	6.21	66	80	0.7987
Measure	66	98	542						54	70	
Error	−7	18	16						12	10	
Relative error			2.95%						22.20%	14.29%	

**Table 3:** Sunscreen C02 GA’s detection results. Through perspective transformation, the projection results of the model on the left and right images are shown in Figure 9(b2–d2). The table’s “Measure” row shows the target’s measurement under the tape measure.

Generation	*x* [mm]	*y* [mm]	*z* [mm]	$\varepsilon_{1}$	$\varepsilon_{2}$	$\varepsilon_{3}$	α	β=αk	$L_{\mathrm{Mj}}$ [mm]	$B_{\mathrm{Mj}}$ [mm]	$F_{j}(\Phi_{M}^{j})$
100	−8	79	743	0.16	0.27	0.45	9.44	8.58	56	97	0.3606
500	−7	90	723	0.11	0.03	0.13	9.75	8.84	54	94	0.5971
1000	−7	90	723	0.11	0.03	0.13	9.75	8.84	54	94	0.5971
Measure	14	83	735	0	0.017	0			58	90	
Error	−21	7	12	0.11	0.013	0.13			−4	4	
Relative error			1.63%						6.90%	4.44%	

**Table 4:** Position (mm), orientation (degrees), and size relative errors. In general model-based methods, it is assumed that the model is the same size as the object with no dimensional errors. Results of studies using PM ratio [20] or stereo vision [32] for size measurements are also included in the table for comparison, but they do not have pose detection.

	$e_{x}$	$e_{y}$	$e_{z}$	$e_{1}$	$e_{2}$	$e_{3}$	ΔL	ΔB
Tomato [20]							3.62%	4.11%
Tomato [32]							7.01%	
Milk [30]	3.89	4.25	57.68	38.74	27.62	42.68		
Tide detergent [30]	1.74	0.74	10.71	1.78	1.64	0.8		
Sugar box [39]	<50	<50	<50	<15	<15	<15		
Ours (pear)	−7	18	16				22.20%	14.29%
Ours (sunscreen)	−21	7	12	12.33	3.11	14.69	6.90%	4.44%

## Data Availability

Data sharing is not applicable to this article, as this study has presented all data.
